# ALDH1-High Ovarian Cancer Stem-Like Cells Can Be Isolated from Serous and Clear Cell Adenocarcinoma Cells, and ALDH1 High Expression Is Associated with Poor Prognosis

**DOI:** 10.1371/journal.pone.0065158

**Published:** 2013-06-06

**Authors:** Takafumi Kuroda, Yoshihiko Hirohashi, Toshihiko Torigoe, Kazuyo Yasuda, Akari Takahashi, Hiroko Asanuma, Rena Morita, Tasuku Mariya, Takuya Asano, Masahito Mizuuchi, Tsuyoshi Saito, Noriyuki Sato

**Affiliations:** 1 Department of Pathology, Sapporo Medical University School of Medicine, Chuo-Ku, Sapporo, Japan; 2 Department of Obstetrics and Gynecology, Sapporo Medical University School of Medicine, Chuo-Ku, Sapporo, Japan; The University of Hong Kong, China

## Abstract

Cancer stem-like cells (CSCs)/cancer-initiating cells (CICs) are defined as a small population of cancer cells that have high tumorigenicity. Furthermore, CSCs/CICs are resistant to several cancer therapies, and CSCs/CICs are therefore thought to be responsible for cancer recurrence after treatment and distant metastasis. In epithelial ovarian cancer (EOC) cases, disease recurrence after chemotherapy is frequently observed, suggesting ovarian CSCs/CICs are involved. There are four major histological subtypes in EOC, and serous adenocarcinoma and clear cell adenocarcinoma are high-grade malignancies. We therefore analyzed ovarian CSCs/CICs from ovarian carcinoma cell lines (serous adenocarcinoma and clear cell adenocarcinoma) and primary ovarian cancer cells in this study. We isolated ovarian CSCs/CICs as an aldehyde dehydrogenase 1 high (ALDH1^high^) population from 6 EOC cell lines (3 serous adenocarcinomas and 3 clear cell adenocarcinomas) by the ALDEFLUOR assay. ALDH1^high^ cells showed greater sphere-forming ability, higher tumorigenicity and greater invasive capability, indicating that ovarian CSCs/CICs are enriched in ALDH1^high^ cells. ALDH1^high^ cells could also be isolated from 8 of 11 primary ovarian carcinoma samples. Immunohistochemical staining revealed that higher ALDH1 expression levels in ovary cancer cases are related to poorer prognosis in both serous adenocarcinoma cases and clear cell adenocarcinoma cases. Taken together, the results indicate that ALDH1 is a marker for ovarian CSCs/CICs and that the expression level of ALDH1 might be a novel biomarker for prediction of poor prognosis.

## Introduction

Ovarian cancer is a malignant disease with high mortality and is the fourth most common cause of cancer-related death in women worldwide. [1], [2] Obscure and unclear symptoms make detection of ovarian cancer in the early stage difficult. [3] Generally, ovarian cancer is relatively sensitive to first-line chemotherapy based on platinum/taxane. [4] Clinical complete response (CR) can often be achieved by cytoreductive surgery and chemotherapy in advanced ovarian cancer patients; however, the majority of patients with advanced stage have disease recurrence which is the reason for the high mortality of this disease. [5] Some therapeutic candidates of molecular target drugs for ovarian cancer had been tested, but notable improvements in prognosis were not achieved. [2], [6].

Recent progress in cancer research has revealed that cancers are composed of a heterogeneous population of cells and that only a small population of cells called cancer stem-like cells (CSCs)/cancer-initiating cells (CICs) have high tumor-initiating potential (cancer stem cell hypothesis). CSCs/CICs are defined as a small population of cells that have (1) high tumorigenicity, (2) multiple differentiation ability and (3) self-renewal capability. [7]–[9] Results of recent study have also shown that CSCs/CICs are related to cancer recurrence and resistance to radiation or chemotherapy. [10], [11] Therefore, CSCs/CICs are thought to be responsible for cancer recurrence and distant metastasis, and elimination of CSCs/CICs is therefore indispensable for curing cancer.

There are several approaches for identifying CSCs/CICs from cancers in a variety of organ tissues. [12] These approaches include (1) use of cell surface marker such as CD44^+^CD24^−/low^ESA^+^ [13], CD133^+^ [14], CD44^+^CD117^+^ [15], and CD166^+^ [16], (2) side population (SP) assay [17], in which the cell population that has the ability to pump out a drug (Hoechst33342 [18] or Dye Cycle Violet [19]) through the ATP-binding cassette transporter is regarded as CSCs/CICs, and (3) ALDEFLUOR assay based on the level of aldehyde dehydrogenase 1(ALDH1) enzyme activity. [20].

The function of intracellular ALDH is to catalyze the oxidation of aldehyde, and ALDH therefore plays an important role in cellular homeostasis. Recent studies have revealed that both normal and cancer cells with high levels of ALDH1 activity have the potential to function as stem cells and potentials for self-renewal capability and stress-resistant properties. [20]–[22].

Also in epithelial ovarian cancer (EOC), some researches have suggested the usefulness of ALDH1 activity to identify CSCs/CICs. The existence of cells with high ALDH activity (ALDH1high cells, compared with ALDH1low cells) has also been shown in EOC cell lines and in clinical specimens. [23]–[25] The correlation between ALDH1 activity and prognosis of patients, however, is still controversial in EOC. [26] At the same time, the relevance to ALDH1 expression for each histological subtype of EOC has not been clarified yet.

In this study, we identified and evaluated the stemness of ALDH1^high^ cells in serous adenocarcinoma and clear cell adenocarcinoma of the ovary, not only from established cell lines but also from primary ovarian cancer cells. We also statistically analyzed the association between ALDH1 expression and clinical outcome for ovarian cancer patients.

## Materials and Methods

### Ethics Statement

Mice were maintained and experimented on in accordance with the guidelines of and after approval by the Committee of Sapporo Medical University School of Medicine, Animal Experimentation Center under permit number 08-006. Any animal found unhealthy or sick was promptly euthanized. All studies were approved by Institutional Review Boards (IRB) of Sapporo Medical University Hospital and the IRB of Hakodate Goryokaku Hospital. Written Informed consent was obtained from all patients according to the guidelines of the Declaration of Helsinki.

### Cell Lines and Culture Conditions

In this study, 3 ovarian serous adenocarcinoma cell lines (AMOC-2, HUOA and OVCAR-3) and 3 ovarian clear cell adenocarcinoma cell lines (ES-2, RMG-1 and TOV21G) were used. AMOC-2 (kindly provided by Dr. Yabushita, Department of Obstetrics and Gynecology, Aichi- Medical University, Aichi, Japan) [27], HUOA (kindly provided by Dr. Ishiwata, Obstetrics and Gynecologic Hospital, Ibaraki, Japan) [28], OVCAR-3 and TOV-21G (ATCC, Manassas, VA, USA) were cultured in RPMI1640 (Sigma-Aldrich, St Louis, MO, USA). ES-2 cells (ATCC) were cultured in Dulbecco’s Modified Eagle’s medium (DMEM, Sigma-Aldrich). RMG-1 cells (JCRB Cell Bank, Osaka, Japan) were cultured in DMEM/F-12 (Life Technologies, Grand Island, NY, USA). Each medium was supplemented with 10% fetal bovine serum (FBS). Cells were incubated in a humidified 5% CO_2_ incubator at 37°C.

### Isolation of Primary Cancer Cells from Clinical Specimens

All studies were approved by Institutional Review Boards (IRB) of Sapporo Medical University Hospital and the IRB of Hakodate Goryokaku Hospital. Written Informed consent was obtained from all patients according to the guidelines of the Declaration of Helsinki.

Solid tumors were cut into fragments, washed in phosphate buffered saline (PBS), and centrifuged at 2000 rpm for 10 minutes. Then cell aggregates were incubated at 37°C for about 30 min with 2 mg Liberase™ research grade (Roche, Basel, Switzerland) in 10 ml Iscove’s Modified Dulbecco’s Medium (IMDM, Life Technologies) until separated into single cells. The cells obtained by these procedures were analyzed by flow cytometry as described below.

### ALDEFLUOR Assay and Isolation by Fluorescence-activated Cell Sorting (FACS)

We used an ALDEFLUOR assay kit (Stem Cell Technologies™, Vancouver, BC, Canada) to determine ALDH1 activity of cells according to the manufacturer’s protocol. Cells were suspended in ALDEFLUOR assay buffer containing 1 µmol/l per 1x10^6^ cells of the ALDH substrate, boron-dipyrromethene-aminoacetaldehyde (BAAA), and incubated for 50 min at 37°C. Each sample was treated with 50 mmol/l of an ALDH-specific inhibitor, diethylaminobenzaldehyde (DEAB), as a negative control. Stained cells were analyzed by BD FACSAria™ II (BD Biosciences, San Jose, CA, USA). Cells were stained with 1 µg/ml of propidium iodide to evaluate their viability prior to analysis.

For isolation of epithelial cells from solid ovarian cancer specimens, we sorted epithelial cancer cells using anti-CD326 (Ep-CAM) APC antibody (BD Biosciences). We used anti-CD326 microbeads (BD Biosciences) and an Automacs system (Miltenyi Biotec, Bergisch Gladbach, Germany) to enrich epithelial cells from malignant ascites of ovarian cancer cases before the ALDEFLUOR assay.

### Cell Cycle Analysis and *in vitro* Cell Grow Analysis

ALDH1^high^ and ALDH1^low^ cells were directly fixed with 70% ethanol after sorting. Then they were resuspended in PBS containing 250 µg/ml RNase A (Sigma-Aldrich) for 30 minutes at 37°C and stained with 50 µg/ml propidium iodide for 10 minutes at 4°C in the dark. Stained cells were analyzed with a FACSCalibur (BD Biosciences), and the data were analyzed using the Mod-Fit cell cycle analysis program.

For *in vitro* cell growth assay, ALDH1^high^ cells and ALDH1^low^ cells isolated from AMOC-2 and RMG-1 cells were seeded into a 6-well plate at 5 ×10^4^ cells per well. After incubation for 48 and 96 hours, the cells were removed by trypsin and viable cell numbers were determined using Countess® (Life Technologies).

### Sphere-forming Assay/single Cell Sphere-forming Assay

ALDH1^high^ and ALDH1^low^ cells were isolated by FACS and then cultured in 6-well ultra-low attachment surface dishes (Corning, Tewksbury, MA, USA) at 1000 cells per well. For the single cell sphere-forming assay, both ALDH1^high^ and ALDH1^low^ were sorted into 96-well ultra-low attachment dishes (Corning) at a single cell per well. The cells were cultured in stem-cell medium, serum-free DMEM/F12 (Life Technologies) supplemented with N-2 supplement (Life Technologies), 20 mg/ml recombinant human epithelial growth factor (Life Technologies), 10 mg/ml human basic fibroblast growth factor (Sigma-Aldrich), 4 µg/ml heparin (Sigma-Aldrich), 4 mg/ml bovine serum albumin (Life Technologies), 20 g/ml human insulin, zinc solution (Life technologies), and 2.9 mg/ml glucose (Sigma-Aldrich). [29] Morphological change was observed daily under a light microscope for 14 days.

### *In vivo* Tumorigenicity

Sorted ALDH1^high^ and ALDH1^low^ cells were resuspended at 1.0×10^2^, 1.0×10^3^ and 1.0×10^4^ cells in 100 µl PBS and Matrigel (BD Biosciences) mixture (1:1). Then each mixture was injected subcutaneously into the right/left middle back areas of 6-week-old female non-obese diabetic/severe combined immune-deficiency (NOD/SCID) mice (NOD.CB17-Prdkcscid/J, Charles River Laboratory, Yokohama, Japan) under inhalation anesthesia by isoflurane. Tumor initiation and progression were observed weekly until the mice were sacrificed at 7 weeks after injection. External tumor volume was calculated as 0.5× Dmax× (Dmin)^2^ [mm^3^] (Dmax : long axis, Dmin : short axis of mass).

### Immunohistochemical Staining for Ovarian Cancer Tissue

Immunohistochemical staining of ALDH1 and Ki-67 was performed with formalin-fixed, paraffin-embedded sections of 123 epithelial ovarian cancer tissues (62 serous adenocarcinomas, 37 clear cell adenocarcinomas, 18 endometrioid adenocarcinomas and 6 mucinous adenocarcinomas) as described previously. [30] [31] Antigen retrieval was done by boiling sections in 120°C for 5 min in a microwave oven in preheated 0.01 mol/l sodium citrate (pH 6.0). Endogenous peroxidase activity was blocked by 3% hydrogen peroxide in ethanol for 10 min. After blocking with 1% non-fat dry milk in PBS (pH 7.4), the sections were reacted with mouse anti-ALDH1 monoclonal antibody (1:250, Sigma-Aldrich) or mouse anti-Ki-67 monoclonal antibody (1:100, DAKO, Glostrup, Denmark) for 1 hour followed by incubation with biotinylated anti-mouse IgG (Nichirei) for 30 min. Subsequently, the sections were stained with setreptavidin-biotin complex (Nichirei Biosciences, Tokyo, Japan), followed by incubation with 3,3′–diaminobenzidine used as a chromogen and counter-staining with hematoxylin. Cytoplasmic staining was regarded as positive for ALDH1, and nuclei staining were regarded as positive for Ki-67. For evaluation of ALDH1 staining, the cases were divided into two groups (ALDH1^high^ group and ALDH1^low^ group) by medians (medians for serous adenocarcinoma the median and clear cell adenocarcinoma being 20% and 15%, respectively).

### Matrigel Invasion Assay

The invasive capability of cells was evaluated using matrigel invasion chambers (BD Biosciences). Isolated ALDH1^high^ and ALDH1^low^ cells (1.0×10^4^ ) were plated in each upper chamber in serum-free DMEM. The outer chambers were filled with DMEM including 10% FBS as a chemoattractant. Cells were incubated for 48 hours, and invasive cells were stained with Hematoxylin, mounted on slides, and counted at 400-fold upper field by light microscopy.

### Statistical Analysis

Statistical analysis, data fitting and graphics were performed using SPSS software package ver.19 (SPSS, Chicago, IL, USA). Data are shown as mean ± *SD* of at least 3 independent experiments, and Student’s t-test was used to assess statistically significant differences (p<0.05). Overall survival (OS), defined as interval from the date of first diagnosis to the date of death from disease progression, and progression-free survival (PFS), defined as the interval from the date of first diagnosis to the date of disease progression, were estimated using the Kaplan-Meier method and compared using the log-rank test. Associations of ALDH1 expression with clinical stage, lymph node metastases and dissemination were analyzed by Fisher’s test.

## Results

### Isolation of ALDH1^high^ Cells from EOC Cell Lines

**Figure 1:**
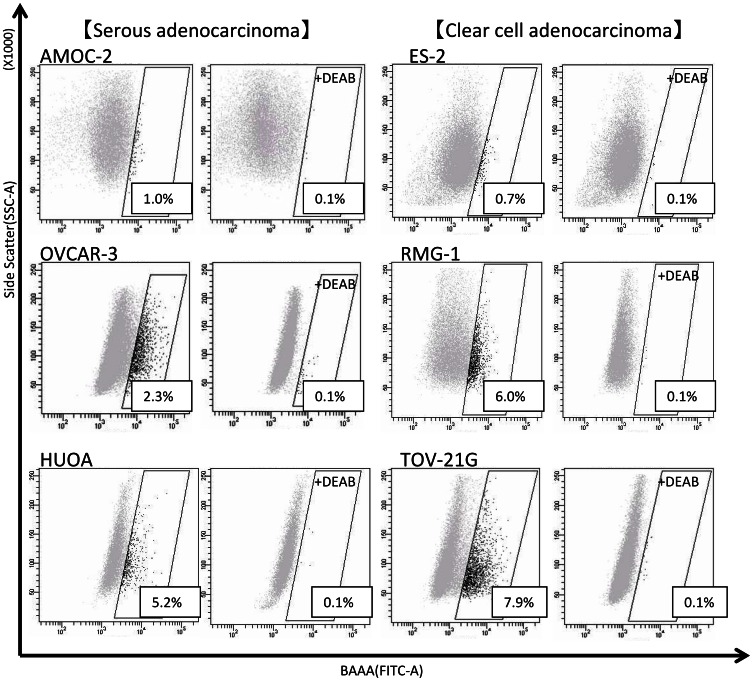
ALDEFLUOR assay for 6 epithelial ovarian cancer cell lines. ALDH1^high^ cells were detected in 3 serous adenocarcinoma cell lines (AMOC-2, HUOA and OVCAR-3) and in 3 clear cell adenocarcinoma cell lines (ES-2, RMG-1 and TOV-21G). SSC-A: single strand conformation analysis. BAAA: boron-dipyrromethene- aminoacetaldehyde. FITC-A: fluorescein isothiocyanate analysis. Percentages in boxes indicate ALDH1^high^ cell ratios. Diethylaminobenzaldehyde (DEAB), an ALDH-specific inhibitor, was used as a negative control.

Several methods to isolate CSCs/CICs have already been described [12], and an aldehyde dehydrogenase 1 high population (ALDH1^high^) identified by the ALDEFLUOR assay was described to be enriched with CSCs/CICs. [20] We therefore analyzed ovarian carcinoma cell lines by the ALDEFLUOR assay to isolate ovarian CSCs/CICs. We investigated 3 human ovarian serous adenocarcinoma cell lines (AMOC-2, HUOA and OVCAR-3) and 3 human clear cell adenocarcinoma cell lines (ES-2, RMG-1 and TOV-21G) (Figure 1). ALDH1^high^ population was identified in all ovarian carcinoma line cells, and the ratio of ALDH1^high^ cells ranged from 0.7% for ES-2 cells to 7.9% for TOV-21G cells. We could isolate ALDH1^high^ cells stably from 4 cell lines (AMOC-2, ES-2, RMG-1 and TOV-21G), and we therefore used these cell lines for further analysis.

### Characterization of ALDH1^high^ Cells

**Figure 2:**
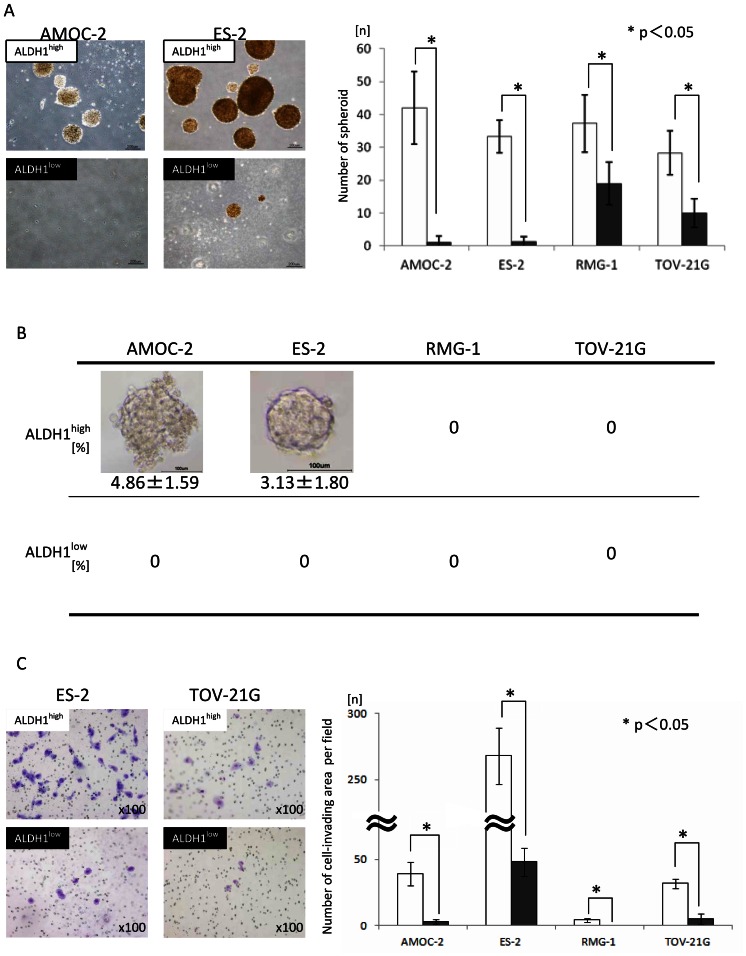
Comparison of sphere-forming ability and invasion capability of ALDH1^high^ cells and ALDH1^low^ cells. **A Sphere-forming assay** One thousand ALDH1^high^ cells and ALDH1^low^ cells were cultured in a floating condition. The spheres were counted on day 10. Magnification of images: ×100. Criterion for spheroid size: over 100µm. Each value is the mean number of spheroids± SD. *P values. **B Single cell sphere-forming assay** Sorted ALDH1^high^ cells and ALDH1^low^ cells were cultured in a floating condition at a single cell per well. The spheres were counted on day 12. Only ALDH1^high^ cells from AMOC-s and ES-2 cells initiated single cell spheroids. Magnification of images: ×200. Each values is the mean percentage of spheroids ± SD. **C Matrigel invasion assay** Images: Matrigel-invading cells derived from ALDH1^high/low^ cells of ES-2 and TOV-21G cells. Magnification of images: ×100. Each values is the mean number of invading cells ± SD. *P values.

Since CSCs/CICs are known to form spheres in floating culture conditions [29], we analyzed ALDH1^high^ cells by a sphere-forming assay. A total of 10^3^ cells per well were sorted and incubated into a 6–well plate in an anchorage-independent environment. At day 10, ALDH1^high^ cells derived from AMOC-2 cells showed greater sphere-forming ability than that of ALDH1^low^ cells. Similar results were obtained from ES-2 cells, RMG-1 cells and TOV-21G cells (Figure 2A). Since the sphere-forming assay is not suitable for quantification of the ratios of CSCs/CICs in ALDH1^high^ cells, we performed a single cell sphere-forming assay. Sphere formation was observed in 4.86% of the wells of ALDH1^high^ cells derived from AMOC-2 cells and in 3.13% of the wells of ALDH1^high^ cells derived from ES-2 cells (Figure 2B). On the other hand, wells of ALDH1^low^ cells derived from both AMOC-2 and ES-2 cells did not show any sphere formation.

We then investigated the invasion ability of ALDH1^high^ and ALDH1^low^ cells by the matrigel invasion assay. ALDH1^high^ cells derived from all four line cells showed greater matrigel invading ability than did ALDH1^low^ cells (Figure 2C).

**Figure 3:**
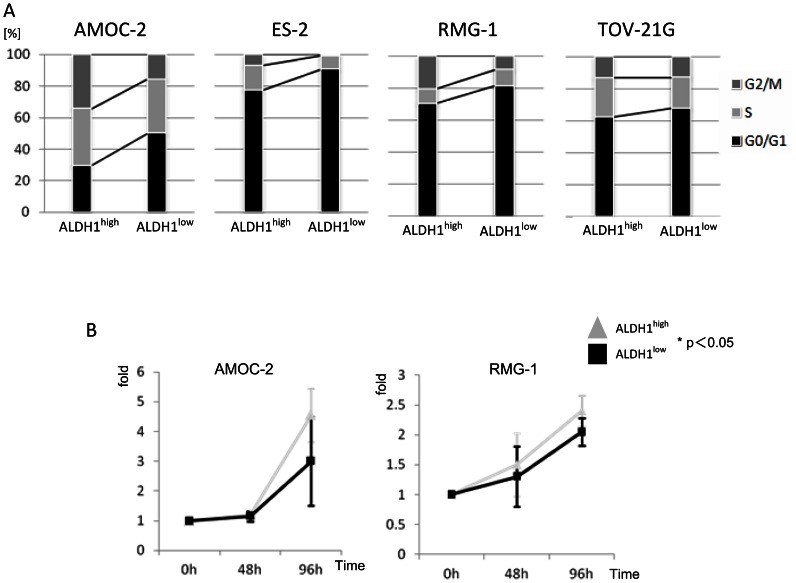
Comparison of cell cycle and growth of ALDH1^high^ cells and ALDH1^low^ cells. **A Cell cycle analysis** ALDH1^high^ and ALDH1^low^ cells were analyzed by a cell cycle assay. The graph indicates the ratios of cells in G0/G1 phase, S phase and G2/M phase. **B Cell growth analysis** The growth capabilities of ALDH1^high^ cells and ALDH1^low^ cells were investigated. Each values is the mean number of cells ± SD.

The cell cycle status of ALDH1^high^ cells and that of ALDH1^low^ cells were analyzed. The ratios of ALDH1^high^ cells in S phase and G2/M phase were greater than ratio of ALDH1^low^ cells in S phase and G2/M phase (Figure 3A). Cell grow analysis revealed that ALDH1^high^ cells derived from AMOC-2 and RMG-1 cells had higher grow rates than those of ALDH1^low^ cells (Figure 3B).

### Higher Tumorigenicity of ALDH1^high^ Cells than that of ALDH1^low^ Cells

**Figure 4:**
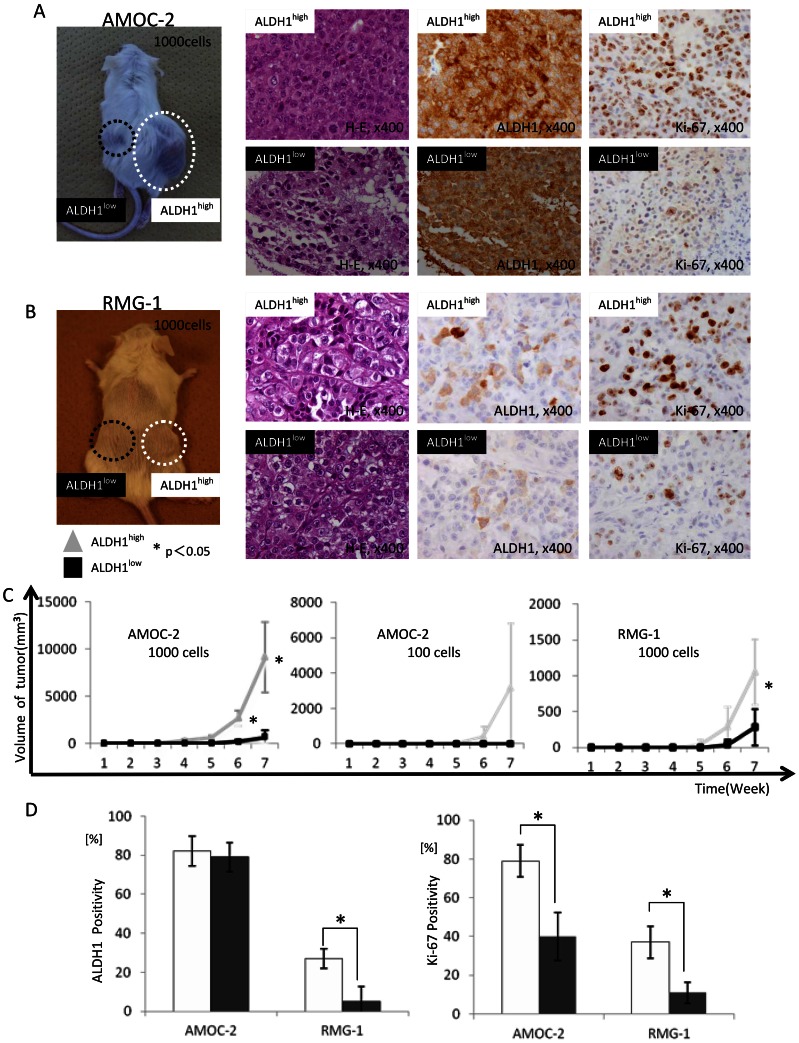
Comparison of in vivo tumorigenicity of ALDH1^high^ cells and ALDH1^low^ cells. **A ALDH1^high/low^ tumors derived from AMOC-2 cells.** **B ALDH1^high/low^ tumors derived from RMG-1 cells.** Picture: NOD/SCID mice that were injected with 1.0×10^3^ ALDH1^high/low^ cells and in which tumors observed on both sides of their back. Histological images: Hematoxylin-Eosin staining (H-E, left panel), ALDH1 immunohistochemical staining (middle panel), Ki-67 immunohistochemical staining (right panel) of ALDH1^high/low^ tumors. Magnification of images: × 400. **C Growth curves of ALDH1^high/low^ tumors derived from AMOC-2 and RMG-1 cells.** Left column: AMOC-2 cells, 1.0×10^3^ injection. Middle column: AMOC-2 cells,1.0×10^2^ injection. Right column: RMG-1 cells, 1.0×10^3^ injection. Each value is the mean tumor volume ± SD. *P values. **D Immunoreactivity to ALDH1 or Ki-67 of ALDH1^high/low^ tumors derived from AMOC-2 and RMG-1cells** Each value is the mean positive percentage ± SD. *P values.

To address the *in vivo* tumorigenicity of ALDH1^high^ and ALDH1^low^ cells from EOC cell lines, xenograft transplantation into NOD/SCID mice by ALDH1^high^ and ALDH1^low^ cells derived from AMOC-2 and RMG-1 cells was performed (Figure 4A, 4B and 4C). As shown in Table 1, tumors were observed in 5 of 5 mice (10^4^ cells injection), 4 of 5 mice (10^3^ cells injection) and 2 of 5 mice (10^2^ cells injection) in which xenotransplantation of ALDH1^high^ cells derived from AMOC-2 cells was performed. On the other hand, tumors were observed in only 3 of 5 mice (10^4^ cells injection) and 2 of 5 mice (10^3^ cells injection) in which xenotransplantation of ALDH1^low^ cells was performed. Furthermore, growth rate of tumors derived from AMOC-2 ALDH1^high^ cells was significantly faster than that of tumors derived from AMOC-2 ALDH1^low^ cells (Figure 4C). Similar results were obtained for RMG-1 cells (Figure 4B and 4C).

**Table 1:** Tumor-initiation incidence of ALDH1^high/low^ cells derived from AMOC-2 and RMG-1 cells.

	AMOC-2		RMG-1	
Number of cells injected into mice	ALDH1^high^	ALDH1^low^	ALDH1^high^	ALDH1^low^
1.0×10^4^	5/5	3/5	4/5	2/5
1.0×10^3^	4/5	2/5	3/5	2/5
1.0×10^2^	2/5	0/5	0/5	0/5

The number indicates the incidence of tumor-initiation of NOD/SCID Mice.

Histological aspects of tumors derived from ALDH1^high^ cells and ALDH1^low^ cells that were isolated from AMOC-2 and RMG-1 cells were investigated (Figure 4A and 4B). Tumors derived from AMOC-2 ALDH1^high^ cells and AMOC-2 ALDH1^low^ cells showed poorly differentiated adenocarcinoma and there was no significant difference. Similarly, tumors derived from RMG-1 ALDH1^high^ cells and RMG-1 ALDH1^low^ cells showed poorly differentiated clear cell adenocarcinoma. Immunohistochemical staining revealed that there was no significant difference in ALDH1-positive rate between tumors derived from AMOC-2 ALDH1^high^ cells and tumors derived from AMOC-2 ALDH1^low^ cells, whereas the tumor derived from RMG-1 ALDH1^high^ cells showed significant higher ALDH1 positive rates than that of tumor derived from RMG-1 ALDH1^low^ cells (Figure 4D). Furthermore, tumors derived from AMOC-2 ALDH1^high^ cells and RMG-1 ALDH1^high^ cells showed significantly higher positive rates for Ki-67 (MIB-1) than those of tumors derived from AMOC-2 ALDH1^low^ cells and RMG-1 ALDH1^low^ cells (Figure 4D).

### Identification of ALDH1^high^ Cells in Primary EOC Samples

**Figure 5:**
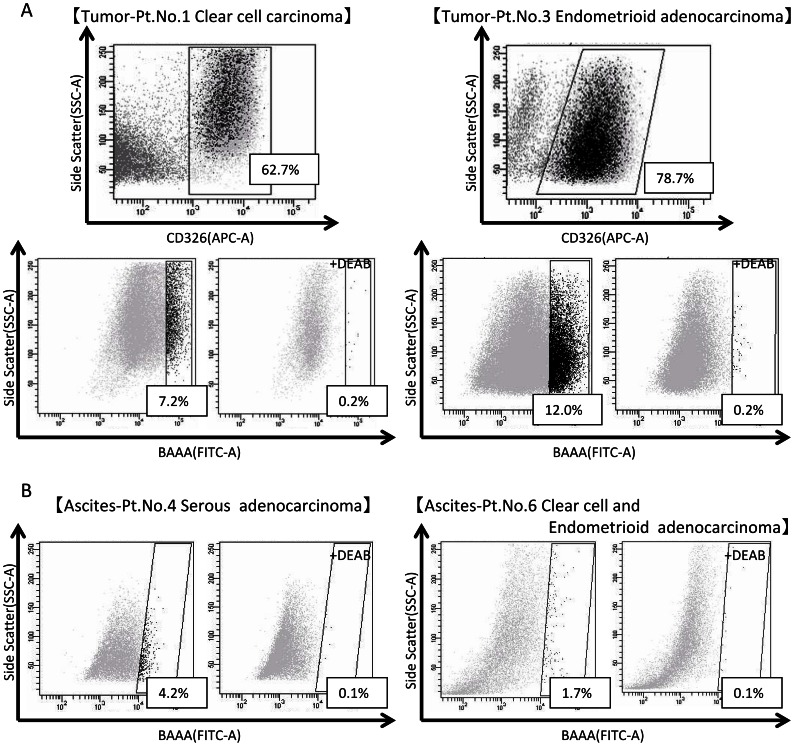
ALDEFLUOR assay for primary solid ovarian cancer cells or cancer cells in ascites. **A Analysis of primary solid ovarian cancer cells** Upper panel shows CD326 staining. Percentage indicat positive rate for CD326 cells. Lower panel shows ALDEFLUOR assay of CD326-positive cells. Left: Patient No.1, case of stage Ic clear cell adenocarcinoma. Right: Patient No.3, case of stage Ic endometrioid adenocarcinoma. **B Analysis of primary cancer cells from ascites** CD326-positive selection was done before ALDEFLUOR analysis. Left: Patient No.4, case of stage IIIc serous adenocarcinoma. Right: Patient No.6, case of stage IIIb clear cell and endometrioid adenocarcinoma. CD326: Epithelial cell adhesion molecule (EpCAM). APC: allophycocyanin area. Percent scores in boxes indicate CD326-positive or ALDH1-activity ratios.

To detect ALDH1^high^ cells in primary ovarian cancer, we analyzed eleven clinical materials using the ALDEFLUOR assay (5 cases of solid ovarian cancer cells and 6 cases of malignant ascites of ovarian cancer cases, summarized in Table 2). CD326-positive epithelial cells were identified in solid ovarian cancer tissues, and the positive rates ranged from 8.1% to 81.0% (Figure 5A, upper panels). ALDH1^high^ cells were detected in 4 of the 5 cases, and positive ALDH1^high^ cell rates ranged from 0.9% to 12.0% (Figure 5A, lower panels). Furthermore, ALDH1^high^ cells were detected in CD326-positive cells derived from ovarian cancer ascites in 4 of the 6 cases, and the positive rates of ALDH1^high^ cells ranged from 1.7% to 4.2% (Figure 5B). Therefore, ALDH1 activity determined by using the ALDEFLUOR assay might be a useful approach for isolation of CSCs/CICs from primary ovarian cancer cells.

**Table 2:** Patient list of clinical specimen that were examined by ALDEFLUOR assay.

Cancer cells from solid cancer tissue					
Pt.No.	Age	Histological subtype	Stage	ALDH1^high^	CD326+[%]
1	42	Clear	Ic	7.2	62.7
2	67	Mucinous	IIIc	0	16.5
3	52	Endometrioid	Ic	12	78.7
4	45	Clear	Ic	8.1	8.1
5	71	Serous	IIIc	2.2	80
Mean	55.4			4.46	49.2
±SD	±13.0			±5.05	±34.5
Cancer cells from ascites					
Pt.No.	Age	Histological subtype	Stage	ALDH1^high^	
1	65	Unknown	IIIc	0	
2	62	Serous	IIIc	2.2	
3	67	Mucinous	IIIc	0	
4	58	Serous	IIIc	4.2	
5	52	Serous	IIIc	4.1	
6	59	Clear+Endometrioid	IIIb	1.7	
Mean	60.5			2.03	
±SD	±5.39			±1.86	

Cancer cell from primary cancer tumor : 6 cases/Cancer cell from ascites : 5 cases.

### Association of High Expression Level of ALDH1 is with Poor Prognosis

**Figure 6:**
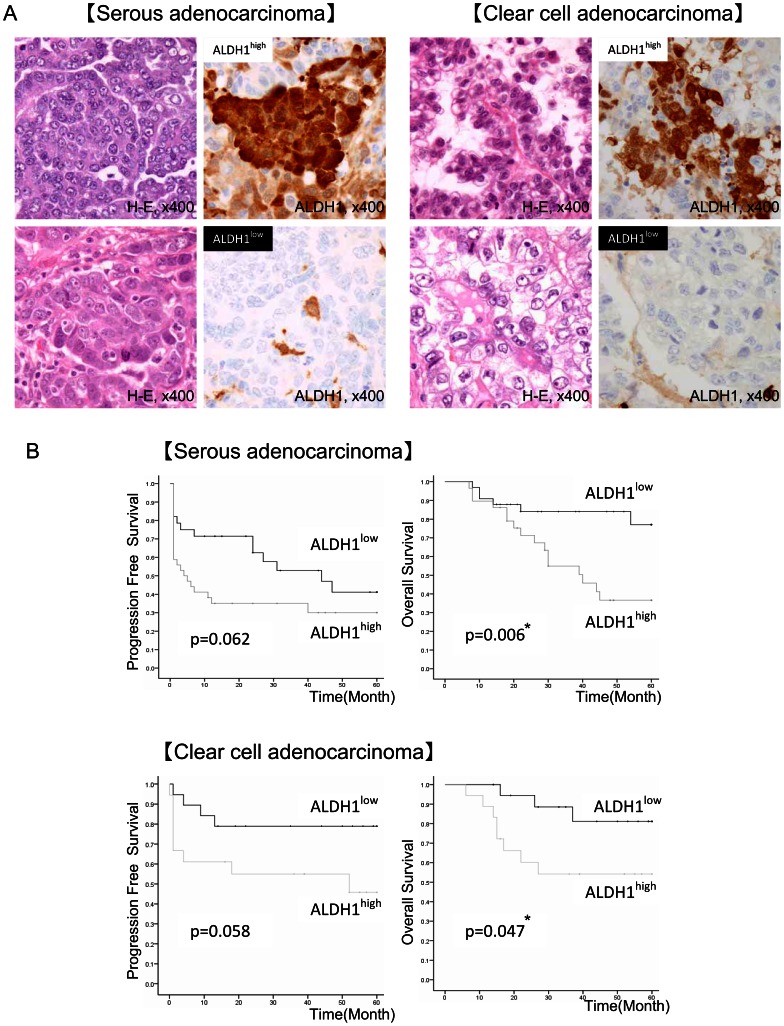
Correlation between ALDH1 immunoreactivity and patients’ clinical outcome in ovarian serous and clear cell carcinoma. **A H-E staining and ALDH1 immunohistochemical staining of primary ovarian serous adenocarcinoma (left) and primary ovarian clear cell adenocarcinoma (right)** Upper left panel: H-E staining of ALDH1^high^ specimen. Upper right panel: ALDH1 immunohistochemical staining of ALDH1^high^ specimen. Lower left panel: H-E staining of ALDH1^low^ specimen. Lower right panel: ALDH1 immunohistochemical staining of ALDH1^low^ specimen. Magnification of images: ×400. **B Log-rank test for ALDH1^high/low^ groups of ovarian serous adenocarcinoma and clear cell adenocarcinoma patients**. Serous adenocarcinoma: 62 cases/clear cell adenocarcinoma: 37 cases. Cases in the ALDH1^high^ group are cases with positive ratio for ALDH1 of over 20% for serous adenocarcinoma cases and 15% for clear cell adenocarcinoma cases. Left: column: progression-free survival (PFS). Right column: overall survival (OS). *P values.

A total of 123 epithelial ovarian cancer tissues were immunohistochemically stained with anti-ALDH1 antibody (Table 3, Figure 6A). The medians of ALDH1-positive rates in serous adenocarcinoma cases and clear cell adenocarcinoma cases were 20% and 15%, respectively. We therefore divided the cases into two groups, ALDH1^high^ group and ALDH1^low^ group, according to the medians. As shown in Table 3, there was no significant correlation of the expression level of ALDH1 with age or with each FIGO clinical stage. High expression level of ALDH1 showed no significant correlation with advanced stages (stage III+IV), T factor or lymph node metastasis in both serous adenocarcinoma cases and clear cell adenocarcinoma cases. The log-rank test revealed that higher expression level of ALDH1 is associated with poorer prognosis with a significant difference in OS of patients with serous adenocarcinoma (P = 0.006) and OS of patients with clear cell adenocarcinoma (P = 0.047) than those of lower expression level of ALDH1 (Figure 6B). Higher expression level of ALDH1 showed a tendency for shorter PFS than that of lower expression level of ALDH1, but the differences were not significant (serous adenocarcinoma: P = 0.062; clear cell adenocarcinoma: P = 0.058).

**Table 3:** Correlation between ALDH1 expression and clinical factors in 123 epithelial ovarian cancer patients.

All cases				
Characteristic	ALDH1^high^	ALDH1^low^	Total	P Value
Patient No.(%)	72(58.5)	51(41.5)	123	
Mean Age ±SD	53.8	50.6	54.8	
[years]	±7.86	±10.7	±10.9	
Pathological				
subtype No.(%)				0.004
Serous	34(54.8)	28(45.2)	62	
Clear cell	18(48.6)	19(51.4)	37	
Endometrioid	14(77.8)	4(22.2)	18	
Mucinous	6(100)	0(0)	6	
FIGO stage No.(%)				0.706
StageI	26(61.9)	16(38.1)	42	
StageII	2(33.3)	4(66.7)	6	
StageIII	39(58.2)	28(41.6)	67	
StageIV	5(62.5)	3(37.5)	8	
Serous adenocarcinoma				
Characteristic	ALDH1^high^	ALDH1^low^	Total	P Value
Patient No.(%)	34(54.8)	28(45.2)	62	
Mean Age ±SD	57.3	55.8	56.6	
[years]	±11.1	±11.9	±11.4	
FIGO stage No.(%)				0.991
I+II	6(60.0)	4(40.0)	10	
III+IV	28(53.8)	24(46.2)	52	
T No.(%)				0.589
T1-2	7(53.8)	6(46.2)	13	
T3	27(55.1)	22(44.9)	49	
Lymphadenectomy				0.537
Case No.(%)	23(53.5)	20(46.5)	43	
L/N Meta (+)				
No. (%)	12(48.0)	13(52.0)	25	
L/N Meta (−)				
No. (%)	11(61.1)	7(38.9)	18	
Clear cell adenocarcinoma				
Characteristic	ALDH1^high^	ALDH1^low^	Total	P Value
Patient No.(%)	18(48.6)	19(51.4)	37	
Mean Age ±SD	50.9	53.0	52.2	
[years]	±8.2	±10.1	±9.4	
FIGO stage No.(%)				0.060
I+II	7(30.4)	16(69.6)	23	
III+IV	8(57.1)	6(42.9)	14	
T No.(%)				0.303
T1-2	14(45.2)	17(54.8)	31	
T3	4(66.7)	2(33.3)	6	
Lymphadenectomy				0.071
Case No.(%)	14(46.7)	16(53.3)	30	
L/N Meta (+)				
No. (%)	4(50.0)	4(50.0)	8	
L/N Meta (−)				
No. (%)	10(45.5)	12(54.5)	22	

P values calculated by Fisher’s test. For all 123 cases, histological subtypes and FIGO clinical stage were investigated about the relevance to ALDH1 expression. Individually in serous or clear cell adenocarcinoma cases, investigated the relevance with ALDH1 expression to FIGO clinical stage, T-classification and lymph nodes metastases.

## Discussion

In this study, we isolated ovary CSCs/CICs with high tumorigenicity by the ALDEFLURO assay. An ALDH1^high^ population could be isolated not only from ovarian cancer cell lines but also from primary ovarian cancer samples. Several methods for isolation of CSCs/CICs have been reported. Indeed, ovarian CSCs/CICs have been successfully isolated by using various methods including the ALDEFLUOR assay [25], side population (SP) analysis [32], [33], and use of CD133^+^ [34], CD44^+^CD24^-^ [35] and CD24^+^ cells. [36] However, there is a controversial report showing that a CSC/CIC marker does not work in some types of cells. [37], [38] Therefore, it is essential to validate the cell population isolated by CSC/CIC markers by several types of analysis. In this study, we confirmed that the ALDH1^high^ population had higher tumorigenicity and greater sphere-forming ability. These observations indicate that the ALDH1^high^ cells we used in this study are enriched with CSCs/CICs. There have been few reports on successful isolation of CSCs/CICs from primly ovarian cancer cells. We isolated ALDH1^high^ cells from 8 of 11 primary ovarian cancer cases. We could not analyze ALDH1^high^ cells from primary ovarian cancers because of the limitation of cell numbers; however, our approach is a possible and promising method for isolating CSCs/CICs from primary ovarian cancers.

Major histological subtypes of EOC are serous adenocarcinoma, clear cell adenocarcinoma, endometrioid adenocarcinoma and mucinous adenocarcinoma, and these subtypes are known to have different characteristics in risk factor of carcinogenesis, molecular biological aspects and so on. More information about individual subtypes is needed to improve the survival of patients. Serous adenocarcinoma is the histology subtype with worst prognosis; however, serous adenocarcinoma cases are often diagnosed at advanced stage. Clear cell adenocarcinoma is gradually increasing up to nearly 12% of EOC in Asian countries, and comparison of the cases in the same stage of 3 histological subtypes (clear cell adenocarcinoma, endometrioid adenocarcinoma and mucinous adenocarcinoma) revealed that clear cell adenocarcinoma is the poorest prognosis in every stages. Therefore, clear cell adenocarcinoma is thought to be the highest grade EOC, recently. [39], [40] Serous adenocarcinoma cases were mainly analyzed in previous studies, and there have been few studies in which clear cell adenocarcinoma cases were analyzed. [23]-[25] In this study, we analyzed not only serous adenocarcinoma cases but also clear cell adenocarcinoma cases. Therefore, these information will bring insight into not only for serous adenocarcinomas, but also most highest grade clear cell adenocarcinomas.

We found that 4 EOC cell lines including 3 clear cell adenocarcinoma lines were positive for sphere formation with 1000 cells/well. On the other hand, only two cell lines (AMOC-2 and ES-2) showed sphere formation in single cell sphere analysis. These results indicate that CSCs/CICs, which have the ability to form a sphere from only one cell, are enriched in ALDH1^high^ cells; however the ratios of CSCs/CICs are not so high even in ALDH1^high^ populations. This result indicated that the ALDEFLUOR assay is just a surrogate marker for CSCs/CICs and that the combination of ALDEFLUOR assay with other markers might be a better approach to isolate CSCs/CICs.

In previous studies on immunohistochemical staining, contradictory results regarding the association of ALDH1 expression with prognosis in EOC were obtained. Chang *et al*. reported that ALDH1 expression correlates with favorable prognosis in serous or non-serous ovarian carcinoma [26], while Deng *et al*. and Wang *et al*. reported opposite results showing that high ALDH1 activity is linked to poor prognosis in serous adenocarcinoma. [23], [25] It should be noted that conditions for immunohistochemical staining and positivity for ALDH1 differed in those studies. In the present study, we set the cut-off lines at 20% for serous adenocarcinoma and 15% for clear cell adenocarcinoma, and we investigated 2 individual histological types. We obtained results showing that high expression level of ALDH1 was associated with poor prognosis in serous and clear cell adenocarcinomas of the ovary. This is the first report showing a relationship between ALDH1 staining and poor prognosis of clear cell adenocarcinomas. On the other hand, ALDH1 expression had no relevance to clinical stage, lymph node metastases or dissemination. These results indicate that ALDH1-positive cells in epithelial ovarian cancer might be responsible for resistance to anti-cancer therapies rather than promotion of diseases. Therefore, ALDH1 might be a novel biomarker for the prediction of prognosis of not only serous adenocarcinoma cases but also clear cell adenocarcinoma cases. Further investigations on molecular aspects of ALDH1^high^ cells in ovarian cancers are needed.

In summary, we successfully isolated an ALDH1^high^ cell population with high tumorigenicity not only from serous adenocarcinoma but also from clear cell adenocarcinoma. ALDH1^high^ cells have higher tumorigenicity and greater sphere-forming ability. ALDH1-positive immunohistochemical staining is related to poor prognosis in both serous adenocarcinoma and clear cell adenocarcinoma. These findings indicate that ovarian CSCs/CICs are positive for ALDH1, and further analysis of ALDH1-positive ovarian CSCs/CICs will lead to the establishment of novel strategies for treating ovarian CSCs/CICs.
